# PTEN dephosphorylates AKT to prevent the expression of GLUT1 on plasmamembrane and to limit glucose consumption in cancer cells

**DOI:** 10.18632/oncotarget.13113

**Published:** 2016-11-04

**Authors:** Suratchanee Phadngam, Andrea Castiglioni, Alessandra Ferraresi, Federica Morani, Carlo Follo, Ciro Isidoro

**Affiliations:** ^1^ Laboratory of Molecular Pathology and Nanobioimaging, Department of Health Sciences, Università del Piemonte Orientale “A. Avogadro”, 28100 - Novara, Italy

**Keywords:** glucose, warburg effect, AKT, PTEN, cancer

## Abstract

GLUT1 is the facilitative transporter playing the major role in the internalization of glucose. Basally, GLUT1 resides on vesicles located in a para-golgian area, and is translocated onto the plasmamembrane upon activation of the PI3KC1-AKT pathway. In proliferating cancer cells, which demand a high quantity of glucose for their metabolism, GLUT1 is permanently expressed on the plasmamembrane. This is associated with the abnormal activation of the PI3KC1-AKT pathway, consequent to the mutational activation of PI3KC1 and/or the loss of PTEN. The latter, in fact, could antagonize the phosphorylation of AKT by limiting the availability of Phosphatidylinositol (3,4,5)-trisphosphate. Here, we asked whether PTEN could control the plasmamembrane expression of GLUT1 also through its protein-phosphatase activity on AKT. Experiments of co-immunoprecipitation and *in vitro* de-phosphorylation assay with homogenates of cells transgenically expressing the wild type or knocked-down mutants (lipid-phosphatase, protein-phosphatase, or both) isoforms demonstrated that indeed PTEN physically interacts with AKT and drives its dephosphorylation, and so limiting the expression of GLUT1 at the plasmamembrane. We also show that growth factors limit the ability of PTEN to dephosphorylate AKT. Our data emphasize the fact that PTEN acts in two distinct steps of the PI3k/AKT pathway to control the expression of GLUT1 at the plasmamembrane and, further, add AKT to the list of the protein substrates of PTEN.

## INTRODUCTION

In cancer cells, glucose is metabolized preferentially through the glycolytic pathway, instead of the mitochondrial oxidative phosphorylation pathway, regardless of the availability of oxygen and in spite of the fact that the glycolytic pathway is more than ten times less convenient in terms of ATP production [1, 2]. Glucose translocation across the plasmamembrane occurs through carriers belonging to the facilitative glucose transporter (GLUT) and the sodium-coupled glucose co-transporter (SGLT) proteins families. While the latter require ATP, the former (GLUTs) allow the glucose entry along the concentration gradient [3]. In humans, fourteen GLUT genes, known as Solute Carrier 2A (SLC2A) 1-14, have been identified and classified in three classes [4]. Of these, GLUT1 is the member with the highest affinity for glucose, and is responsible for the basal uptake of glucose in all tissues. GLUT1 is frequently found upregulated in cancers [5], likely contributing to the avid uptake of glucose even when its availability is becoming insufficient because of the continuous growth of the tumor [3]. GLUT1 expression at the plasmamembrane has been found abnormally high, and to correlate with the malignant features and poor prognosis in tumors from various origin, including prostate [6], thyroid [7, 8], colon [9, 10], melanoma [11], liver [12], breast [13, 14], and ovary [15, 16]. The insertion of GLUTs onto the plasmamembrane occurs through exocytosis of vesicles storing the protein on their membrane [17]. A clear involvement of the PI3k class I (PI3KC1)-AKT pathway and of the AKT downstream effector AS160 (a rab GTPase activator) has been demonstrated in the case of GLUT4 translocation [18, 19]. More recently, we have shown that this pathway is also involved in the cell surface exposure of GLUT1 in thyroid cancer cells [20]. PTEN (Phosphatase and TENsin homolog deleted on chromosome ten), the oncosuppressor protein with dual lipid and protein phosphatase activity, has been shown to contrast the uptake and the large glycolytic consumption of glucose observed in proliferating cancer cells [21]. Indeed, PTEN can inhibit the activation of AKT, thus preventing GLUT1 expression on the plasmamembrane [20]. This effect has been attributed to the lipid phosphatase activity of PTEN that reduces the availability of Phosphatidylinositol (3,4,5)-trisphosphate (PIP3), the phosphate donor for the phosphorylation of AKT. It is unknown whether the protein phosphatase activity of PTEN also plays a role, and if so at which level, in the regulation of GLUT1 translocation onto the plasmamembrane. Here, we demonstrate that PTEN physically interacts with phosphorylated AKT and can efficiently dephosphorylate it. The present findings highlight a novel role of PTEN in the control of GLUT1 expression at the plasmamembrane and, further, add AKT to the list of the substrates of PTEN protein phosphatase.

## RESULTS

### Cell surface expression of GLUT1 in ovarian cancer cells correlates with active AKT

Functional alteration of PI3KC1 and/or of PTEN may cause the hyper-activation of AKT and, consequently, the abnormal expression of GLUT1 on the plasmamembrane seen in cancer cells. Further, we have shown that p53 also controls the expression of GLUT1 at the plasmamembrane [22]. To define the role of the PI3K-AKT pathway and of the oncosuppressors p53 and PTEN in the plasmamembrane expression of GLUT1 in cancer, we have included in our study four ovarian cancer cell lines that differ for the functional expression of these proteins (Table 1). A search on the data base COSMIC (http://cancer.sanger.ac.uk/cell_lines) for genomic mutations of PI3KC1 and AKT, revealed that all the ovarian cancer cell lines under study express wild type AKT, yet only the OVCAR-3 cell line express wild type PI3KC1 (see Table 1). The mutations found in PI3KC1 from OAW42, A2780 and SKOV-3 cells have been reported to activate the AKT-mTOR pathway [23–25]. To be noted, only OAW42 cells express both p53 and PTEN in the wild type (wt) form, A2780 cells express wt p53 and mutated PTEN, SKOV-3 cells express wt PTEN but are p53 null, and OVCAR-3 cells express mutant p53 while the status of PTEN is unknown.

**Table 1:** Summary of the main mutations in the PI3K-Akt pathway and p53, up to now reported in cell lines used in this study (Cosmic v75, http://cancer.sanger.ac.uk/cosmic)

Cell line	PI3KCI	PTEN	Akt	P53
**OAW42**	H1047L	WT	WT	WT
**OVCAR 3**	WT	WT ?	WT	R248Q
**A2780**	E365K	K128_R130del	WT	WT
**SKOV-3**	H1047R	WT	WT	Null

**Figure 1:**
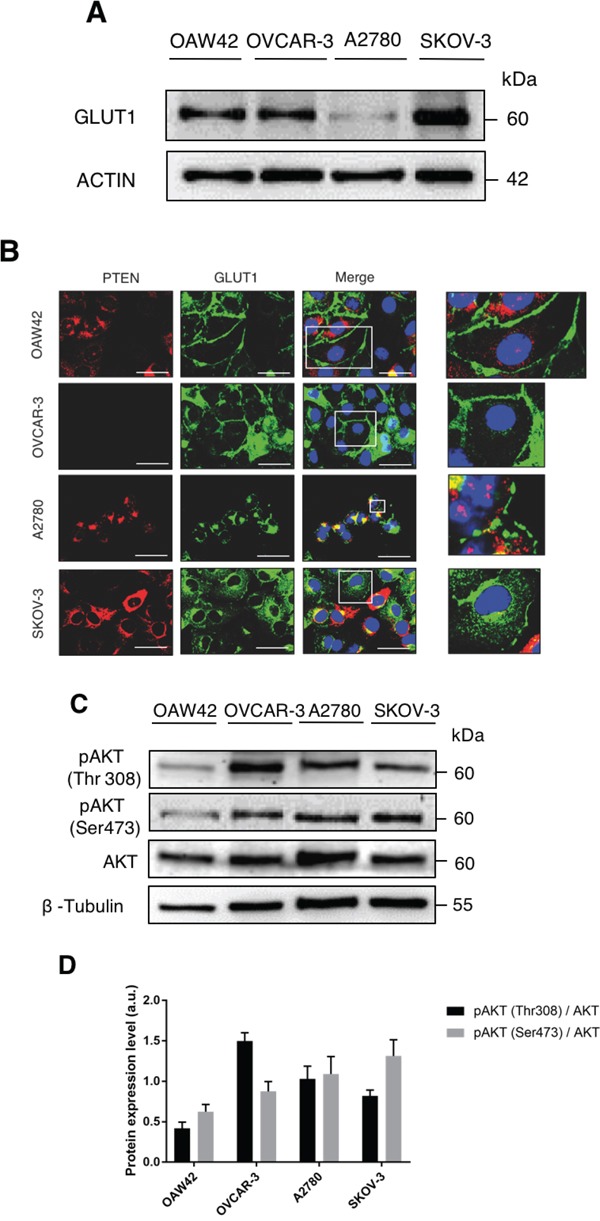
Active AKT promotes cell surface expression of GLUT1 **A.** OAW42, OVCAR3, A2780 and SKOV3 ovarian cancer cells were analyzed by Western blot to reveal the basal expression levels of GLUT-1. β-Actin was used as protein loading marker. One out of four western blotting with similar pattern of protein expression is shown. **B.** The cells were cultured in control media then fixed in ice-cold methanol and co-stained for PTEN and GLUT-1. DAPI (blue) was used to stain nuclei. Scale bar = 20 μM; Magnification = 63X. Representative images of four separate experiments are shown. **C.** OAW42, OVCAR-3, A2780 and SKOV3 cells were plated and cultured in control media. The lysates were analyzed by Western Blot to reveal the expression levels of active AKT, phosphorylated at threonine 308 and serine 473. β-Tubulin was used as protein loading marker. **D.** Densitometric evaluation of phosphorylated AKT levels per each cell line (mean + S.E.M of three separate experiments).

The level of GLUT1 protein expressed in the four ovarian cancer cell lines was analyzed by western blotting. SKOV-3 cells expressed the highest level and A2780 cells expressed the least level of GLUT1 protein (Figure 1A). Next, we looked at the subcellular localization of GLUT1 in these cells. Concomitantly, we also analyzed the expression and subcellular localization of PTEN in the cells. GLUT1 was found mainly at the cell surface in OAW42 and OVCAR-3 cells, it was expressed at low level and distributed partly on the membrane and largely at one pole of the nucleus in A2780 cells, and it was highly expressed and largely diffused in the cytoplasm in SKOV-3 cells (Figure 1B). PTEN was highly expressed in the majority of SKOV-3 cells, it was mainly concentrated at one pole of the nucleus in A2780 cells (where it largely co-localized with GLUT1), it was expressed only in a minority of OAW42 cells, and it was undetectable in OVCAR-3 cells (Figure 1B). In searching for the possible correlation of GLUT1 membrane expression and the activation of the AKT pathway, we assessed by western blotting the phosphorylation status of the AKT protein in the four cell lines. AKT is phosphorylated at Threonine 308 by PDK1 and at Serine 473 by MTORC2. Both these sites were phosphorylated in AKT from all cell lines (Figure 1C-1D). OVCAR-3 cells showed, however, the highest level of basal phosphorylation (especially at Thr308) of AKT.

### Glucose uptake in ovarian cancer cells correlates with PTEN protein expression

**Figure 2:**
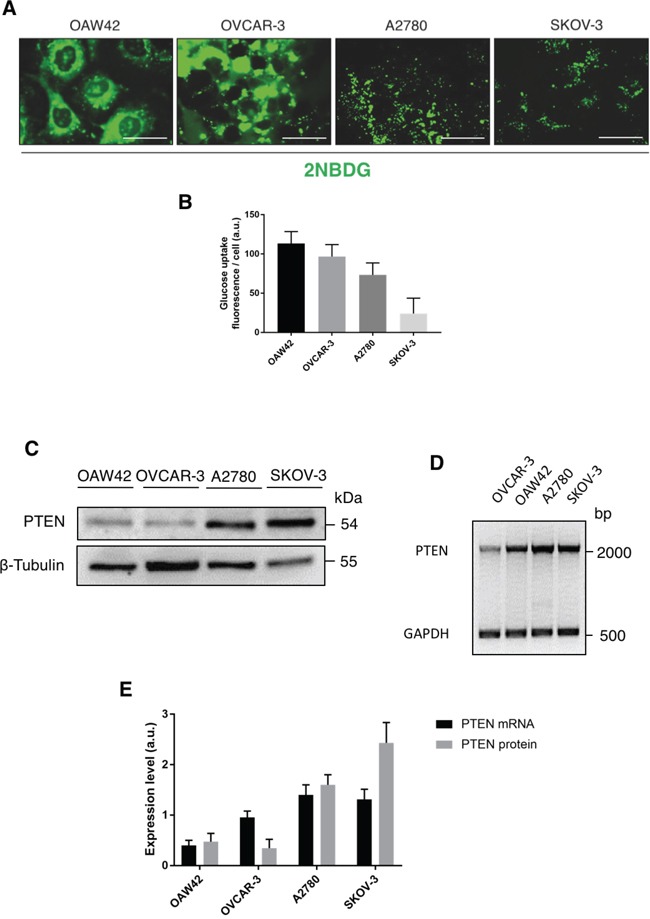
PTEN is directly involved in the regulation of glucose uptake **A.** Cells were incubated for 1 hour with the fluorescent glucose analog probe 2-NBDG 50 μM, then the images were acquired immediately by fluorescence microscopy. Scale bar = 20 μM; Magnification = 63X. **B.** ImageJ quantification of the glucose uptake (average fluorescence intensity per cell) in the various cell lines. Five randomly chosen fields per sample were evaluated for a minimum of hundred cells. Data represented as mean + S.E.M. of four separate experiments. **C.** The cell lysates were analyzed by Western blotting to reveal the expression level of PTEN. A similar pattern of protein expression was reproduced in three other experiments. **D.** Representative agarose gel electrophoresis of PTEN specific mRNA obtained by RT-PCR amplification. GAPDH was used as house-keeping control and co-amplified in the same PCR reaction. **E.** Quantitation of PTEN mRNA and protein levels normalized to the respective house-keeping control.

As a readout of the functional role of GLUT1 at plasmamembrane level, we monitored the uptake of glucose using 2-NBDG, a fluorescent glucose analogue that is internalized through the glucose transporters [26]. Glucose was greedily internalized in OAW42 and OVCAR-3, while it was much less efficiently internalized in A2780 and SKOV-3 (Figure 2A-2B), in perfect agreement with the level of GLUT1 expression at the cell surface (Figure 1B). We notice that the uptake of glucose is negligible in SKOV-3 cells, in spite of the facts that these cells express a mutant active PI3KC1. Glucose uptake is negligible also in A2780 cells. These cells express, however, very low level of GLUT1 (Figure 1A) that mainly reside in a para-golgian area (Figure 1B). By contrast, glucose is efficiently internalized by OAW42 cells and OVCAR-3 cells. Next, we focused on the expression of PTEN. By western blotting, A2780 and SKOV-3 expressed the highest level of the protein (being mutated in the case of A2780 cells), while both OAW42 and OVCAR-3 cells expressed the least level of the protein (Figure 2B-2C). Further, semiquantitative multiplex RT-PCR data (Figure 2D-2E), indicated that OVCAR-3 cells were the least expressing PTEN mRNA, while A2780 and SKOV-3 cells expressed the highest level of PTEN mRNA (about two-folds that of OVCAR-3 cells), and OAW42 cells expressed PTEN mRNA at a level intermediate between the values in OVCAR-3 and the other cell lines (Figure 2D-2E). The low level of PTEN expression at both protein and mRNA levels in OAW42 and OVCAR-3 cells could well explain why these cells maintain GLUT1 at the plasmamembrane and have the highest rate of glucose uptake. Therefore, we further investigated on the regulation of expression of PTEN and on its role in regulating GLUT1 membrane translocation and glucose uptake in OAW42 and OVCAR-3 cells.

### Epigenetic up-regulation of PTEN limits glucose uptake and cell surface expression of GLUT1 in OAW42 cells

**Figure 3:**
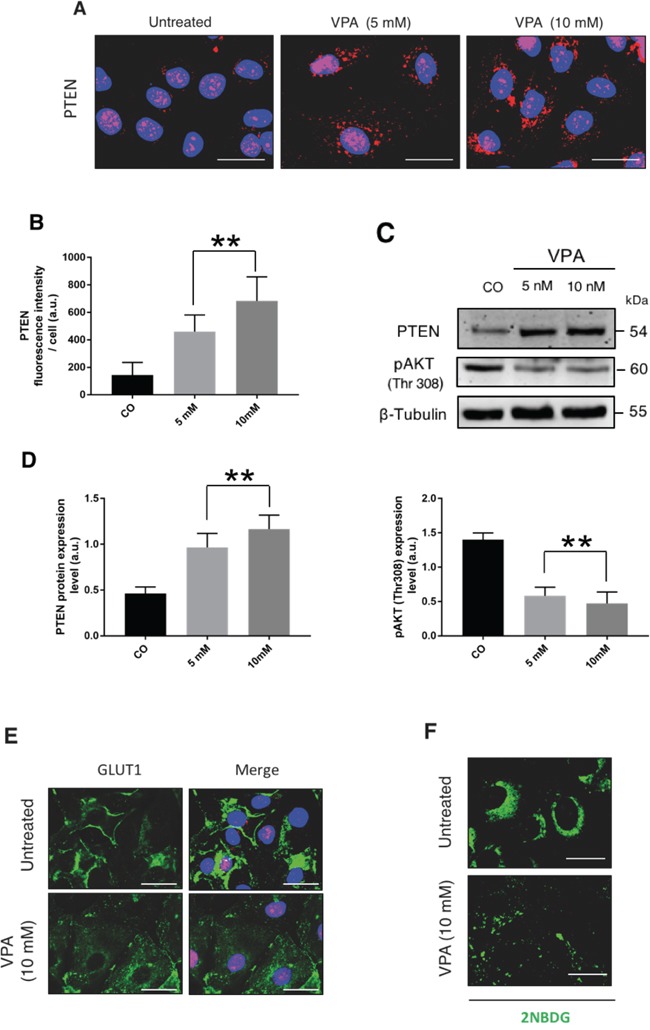
Epigenetic re-expression of PTEN antagonizes glucose uptake in OAW42 **A.** OAW42 cells were treated for 24 h with 5 mM and 10 mM Valproic acid (VPA), a histone deacetylase inhibitor, thereafter the cells were fixed and stained for PTEN. **B.** PTEN expression level increases in VPA-treated cells, as quantitated by fluorescence intensity per cell. **C.** The cell lysates were processed by immunoblotting to reveal PTEN expression along with pAKT (Thr308) as a readout of PTEN activity. A blot representative of three separate experiments is shown. **D.** Densitometric data of the experiment shown in panel C. **E.** OAW-42 cells were treated with VPA 10 mM for 24 h, then stained and imaged under the fluorescence microscope to reveal the localization of GLUT-1. DAPI (blue) was used to counter-stain the nuclei. **F.** OAW-42 cells were treated for 24 h with 10 mM VPA. The fluorescent glucose probe 2NBDG was added 1 h before the end of incubation. Cells were imaged by fluorescence microscope. Scale bars = 20 μM; Magnification = 63X. Images in panels E and F are representative of four separate experiments.

We suspected that in OAW42 cells PTEN could be epigenetically down-regulated. Valproic acid (VPA) is a histone de-acetylase inhibitor that is able to abrogate the epigenetic silencing of PTEN [27, 28]. The incubation with 5 mM or 10 mM VPA for 24 h increased the expression of PTEN protein and induced its relocation from the nucleus to the cytoplasm (Figure 3A-3D). The increase in PTEN expression was paralleled by a reduced level of phospho-AKT at Thr 308 (Figure 3C-3D), of GLUT1 expression at the cell surface (Figure 3E), and of glucose uptake (Figure 3F). Similar data were obtained when OAW42 cells were incubated with 25 μM EpiGalloCatechin-3Gallate (EGCG; not shown), another epigenetic modulator that acts through the inhibition of DNA methyl transferase [29]. On the other hand, when OVCAR-3 cells were incubated with VPA or EGCG for 48 h, the level of PTEN protein remained essentially unchanged (not shown).

### OVCAR-3 cells express the mutant PTEN 464A>G allele

**Figure 4:**
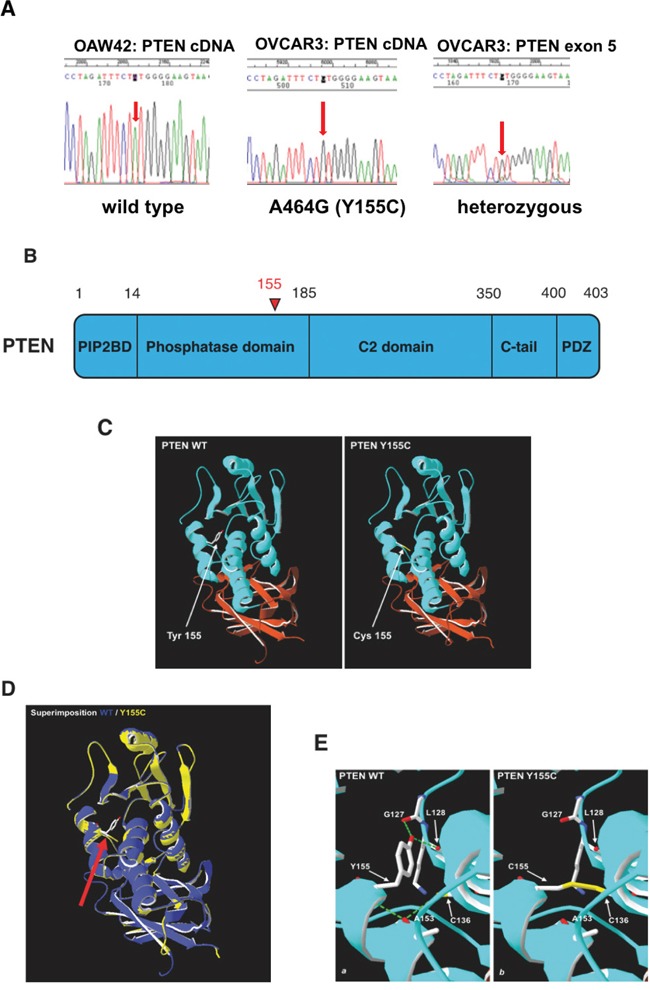
DNA sequencing of PTEN isolated from OVCAR-3 cells **A.** Electropherogram of the PTEN sequence highlighting the portion of cDNA bearing the mutation in OVCAR-3 (middle panel), as compared to the same region of cDNA from OAW42 cells, which express wild-type PTEN (left panel). The sequencing of the PTEN exon 5 of genomic DNA from OVCAR-3 (right panel) indicates that the mutation is effectively present in the germ line (i.e., it is not a PCR artefact) and that it does not occur in the paired allele. **B.** Localizationof the relevant functional domains in the molecule of PTEN. The mutated aminoacid position (155) in the phosphatase domain is indicated in red. **C.** Predicted structure models of WT and Y155C PTEN molecules in which the functional domains are marked in different colors: PIP2 Binding Domain (in green); Phosphatase domain (in blue); C2-domain (in orange); C-tail and PDZ domain (in magentas). In red, marked with an arrow, is indicated the tyrosine mutated in cysteine at position 155. **D.** Overlay of the wild-type (green) and Y155C mutated (red) phosphatase domain predicted structures. The Thyrosine to Cysteine substitution at position 155 (magentas) is pointed by the arrow. **E.** Predicted bonds between amino acids in the wild-type and in the Y155C mutated PTEN structure. *a*) Tyr155 (red) in wild-type PTEN forms H bonds (green) with Gly127 and Lys128; *b*) Cys155, in mutant PTEN forms a disulfide bridge with Cys136, which in wild-type PTEN would form an H-bond with Ala153.

The fact that in OVCAR-3 cells the low expression of PTEN mRNA and protein was not attributable to epigenetic silencing mechanisms, prompted us to investigate for the presence of gene mutation in this oncosuppressor. First, we sequenced the PTEN cDNA isolated from all the cell lines. In A2780 cDNA it was found the 9 bp deletion 381_389del, corresponding to the 3 amino acids deletion K128_R130del (COSMIC), previously reported in literature [30]. Analysis of OAW42 and SKOV-3 cDNAs confirmed that these cell lines express a wt PTEN (COSMIC). Very interestingly, in OVCAR-3 cDNA we found the presence of the nucleotide substitution 464A>G (TAT>TGT), corresponding to the aminoacid substitution Y155C (Figure 4A). To rule out the possibility that this substitution had been artificially introduced in the cDNA by the PCR reaction, we have isolated and sequenced the exon 5 of the genomic DNA from OVCAR-3 cells. Exon 5 sequencing showed the presence of the nucleotide substitution 464A>C along with the wild type exon 5 (represented at very low level), thus confirming that OVCAR-3 cells express the PTEN allele with the missense Y155C mutation (Figure 4A). This mutation locates in the phosphatase domain (Figure 4B), and it has been proven to knock-down the lipid phosphatase activity of PTEN on the Ins(1,3,4,5)P4 substrate [31]. Whether and how the mutation Y155C alters the structure of the protein and affects the dual phosphatase function of PTEN is unknown. To get an insight on this, we have determined the theoretical 3D structure of Y155C mutant PTEN employing Swiss-Model. As shown in Figure 4C, the presence of the substitution Y155C does not alter the 3D structure of PTEN, including that of the phosphatase domain (Figure 4C). Superimposition of the predicted 3D structure of Y155C mutant PTEN and of the crystallographic structure of wt PTEN (1D5R.1.A) (Figure 4D) confirms a high similarity between the structures (RMS=0.87 Å). Analysis of the predicted 3D structure shows that the substitution of the wt Tyrosine 155 with a Cysteine residue leads to the loss of two hydrogen bonds between the wt Tyrosine 155 and the two residues of Glycine 127 and Lysine 128 (Figure 4E). Moreover, according to the predicted structure of the Y155C mutant PTEN, the Cysteine at position 155 forms a disulphide bond with Cysteine 136 and prevents the latter to form a hydrogen bond with Alanine 153.

### The ectopic expression of either the wild type or the protein-phosphatase proficient PTEN, but not of C124S or K128_R130del PTEN, limits the cell surface expression of GLUT1 and the uptake of glucose in OVCAR-3 cells

**Figure 5:**
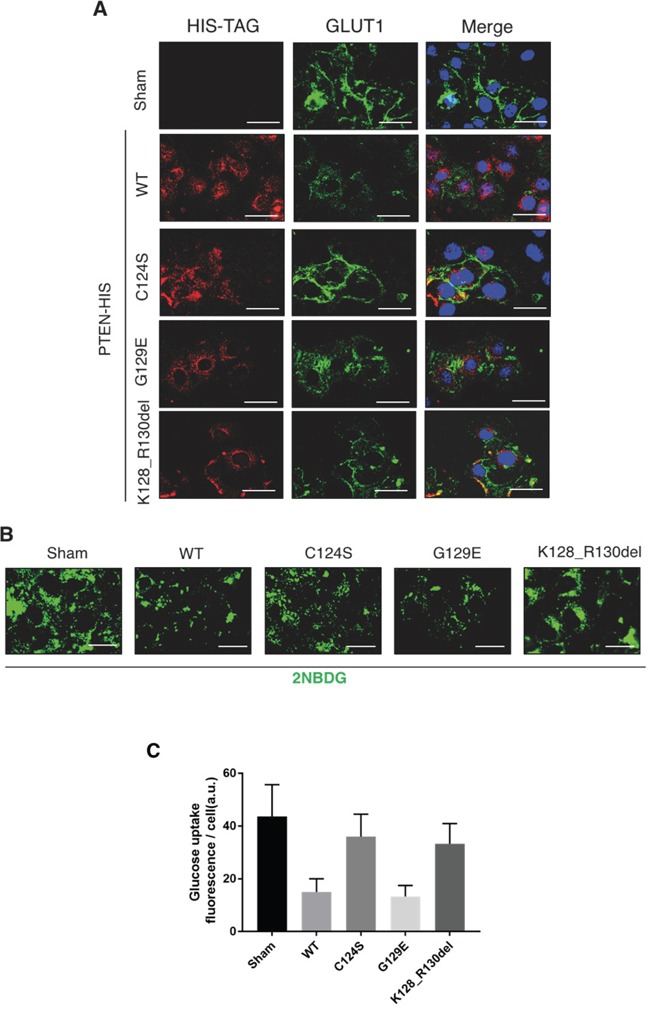
PTEN mutants devoid of protein phosphatase activity cannot prevent the cell surface expression of GLUT1 OVCAR-3 ovarian cancer cells were plated and let adhere onto coverslips. Thereafter, the cells were transfected with empty (Sham), WT PTEN, C124S mutant PTEN, G129E mutant PTEN and Deletion 381-393 mutant PTEN HIS-tagged plasmids. The cells were incubated for at least 36 h post-transfection to allow the synthesis of transgenic PTEN before any treatment. **A.** Cells were fixed in ice-cold methanol and co-stained for HIS-TAG and GLUT-1. DAPI (blue) was used to stain the nuclei. **B.** The cells were incubated for 1 h with the fluorescent glucose analog 2-NBDG (50 μM), then images were acquired by fluorescence microscope. **C.** Fluorescence intensity quantitation of glucose uptake per cell. Scale bar = 20 μM; Magnification = 63X. Representative images of three separate experiments are shown.

**Figure 6:**
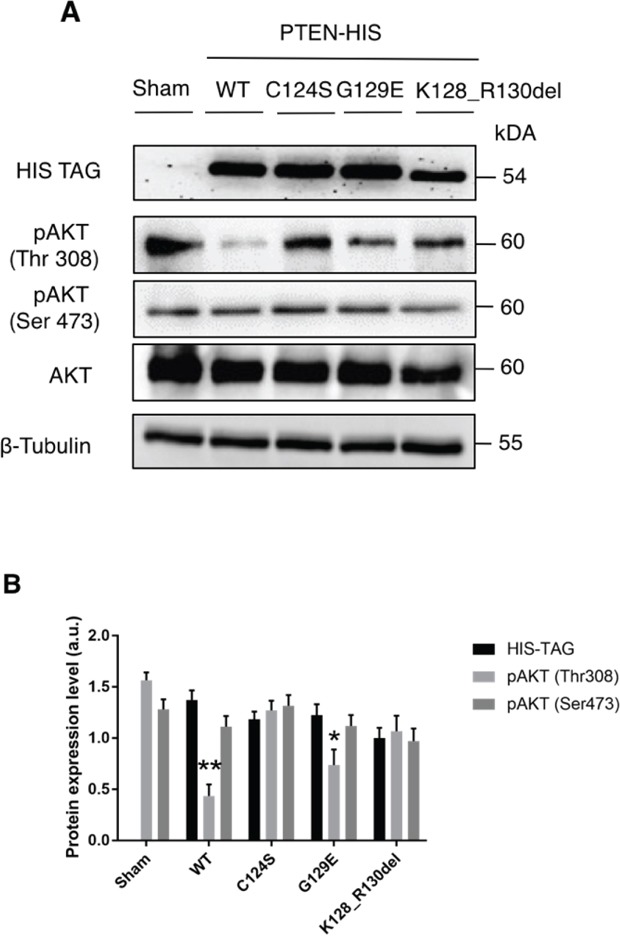
Transgenic re-expression of protein phosphatase endowed PTEN limits the activation of AKT in OVCAR-3 cells **A.** OVCAR-3 ovarian cancer cells were plated and let adhere onto coverslips. Thereafter, the cells were transfected with empty (Sham), WT PTEN, C124S mutant PTEN, G129E mutant PTEN and Deletion 381-393 mutant PTEN HIS-tagged plasmids. The cells were incubated for 36 h post-transfection to allow the synthesis of transgenic PTEN, then the cell lysates were used for Western blotting to reveal the level of expression of transgenic PTEN (anti-HIS), and of active (pAKT at Ser473 and at Thr308) and total AKT. A similar pattern of protein expression was observed in two other separate experiments. **B.** Densitometry analysis (from three separate experiments) of the level of AKT activation in transfected OVCAR-3 cells expressing (or not) PTEN, either wild-type or mutant isoforms as detailed in panel A.

The above data show that OVCAR-3 cells express very low level of PTEN mRNA, which translates into the Y155C mutant protein that lacks of the IP4 lipid-phosphatase activity [31]. This fact could well explain the high expression of GLUT1 on the plasmamembrane and the high glucose uptake in OVCAR-3 cells. To assess the functional role of PTEN in these processes, we expressed individually various isoforms of PTEN in OVCAR-3 cells. Namely, we used the wt, the C124S mutant isoform that lacks both the lipid- and protein-phosphatase activity [32], and the G129E mutant isoform that lacks the lipid-phosphatase activity though still maintaining the protein-phosphatase activity [33, 34]. In the experiment, we have also included the K128_R130del mutant isoform that is expressed in A2780 cells. This mutant is unable to inhibit the activation of the AKT pathway [35], as also shown in Figure 1C. To allow a clear discrimination from endogenous PTEN and to monitor the events specifically in the cells transfected transiently, we have used a plasmid bearing the cDNA for the PTEN isoforms tagged with the epitope HIS [PTEN-(His)_6_]. Image data shown in Figure 5A indicate that in the cells expressing the empty vector (sham), GLUT1 is permanently localized on the cell surface, as shown above. A similar pattern is found in the cells expressing either the C124S or the K128_R130del mutant isoform of PTEN. By contrast, the cells expressing either the wt or the G129E (which only has protein-phosphatase activity) show a prominent cytoplasmic localization of GLUT1. We further confirmed the functional role of PTEN in glucose uptake using the fluorescent analogue 2NBDG. The image data in Figure 5B (and its quantitation shown in Figure 5C) clearly demonstrate that only the expression of the PTEN wt or of the G129E mutant could limit the uptake of glucose, consistent with the absence of GLUT1 at the cell surface of the transfected cells. In a parallel experiment, we have analyzed by western blotting the effect of the ectopic expression of the various PTEN isoforms on the activation of the AKT pathway. The results are shown in Figure 6A-6B. The ectopic over-expression of the C124S or of the K128_R130del mutant isoform of PTEN had no impact on AKT phosphorylation at either Serine 473 or Threonine 308. On the contrary, the phosphorylation at Threonine 308 was greatly reduced, while that at Serine 473 was substantially unaffected, in the cells expressing either the wt or the G129E PTEN isoform.

### The ectopic expression of the protein-phosphatase proficient PTEN is sufficient to limit the activation of AKT in OVCAR-3 cells

**Figure 7:**
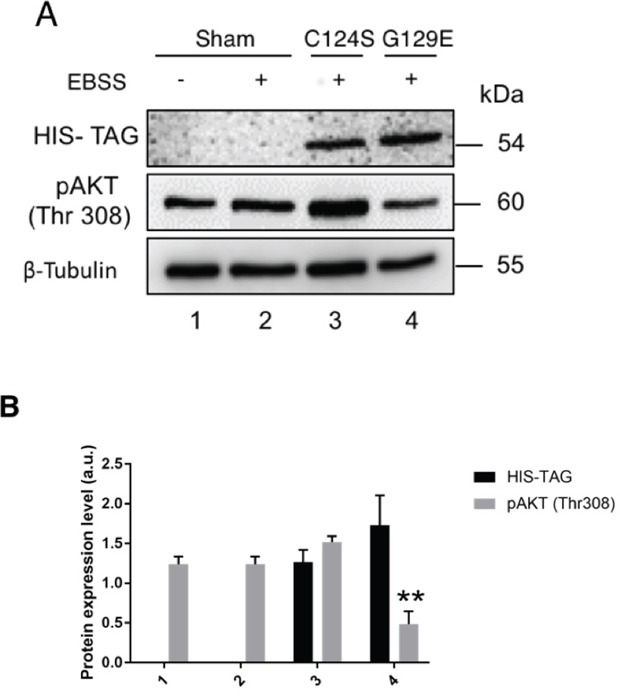
G129E PTEN but not C124S PTEN limits the activation of AKT under starvation conditions **A.** OVCAR-3 cells transfected with empty (Sham), C124S mutant PTEN or G129E mutant PTEN HIS-tagged plasmids. The cells were incubated for 36 h post-transfection to allow the synthesis of transgenic PTEN and then cultured for further 24 h in a medium (EBSS) lacking growth factor (GF) and aminoacids (AA). Thereafter, the cell lysates were used for Western blotting to reveal the level of expression of transgenic PTEN (anti-HIS) and of active (pAKT at Thr308) AKT. A similar pattern of protein expression was observed in two other separate experiments. **B.** Densitometry analysis (from three separate experiments) of the level of AKT activation in transfected OVCAR-3 cells as described in panel A.

Altogether, the above data suggest that the protein phosphatase function of PTEN is of pivotal importance in limiting the plasmamembrane translocation of GLUT1, and this action correlates with the dephosphorylation of AKT at Thr308. While wt PTEN can prevent the activation of AKT by limiting the availability of PIP3 in the cells exposed to growth factors, both the G129E and C124S mutants lack the lipid phosphatase activity. To further discriminate at which step PTEN can affect the phosphorylation of AKT, we have expressed either G129E or C124S PTEN isoform in OVCAR-3 cells and looked at the phosphorylation status of AKT upon incubation of the cells in a culture medium (EBSS) deprived of amino acids (AA) and of serum growth factors (GFs). Although OVCAR-3 cells express a mutant inactive PTEN, theoretically they could express at very low level the normal allele. The latter could counteract the basal activation of the AKT pathway triggered by external stimuli, such as GFs or AA. Note that AA could control the glucose uptake and GLUT1 function by direct activation of mTORC1 [36]. AKT was highly phosphorylated at Thr308 in the cells incubated in the absence of AA and of GF as much as in the cells incubated in complete medium (Figure 7A-7B), suggesting that a normal PTEN is not expressed in these cells and further confirming that the Y155C PTEN mutant is unable to counteract the PI3KC1-AKT pathway. The ectopic expression of C124S-PTEN could not reduce the level of pAKT, even though the cells were incubated in the absence of any external inducer of the PI3K-AKT (mTORC1) pathway (Figure 7). Strikingly, the cells incubated in this condition and expressing the transgenic G129E-PTEN show a very low level of phosphorylation of AKT at the Thr308 site (Figure 7). These data suggest that G129E PTEN, which cannot prevent the PIP3-dependent phosphorylation of AKT because it lacks the lipid-phosphatase activity, may instead reduce the phosphorylation of AKT through a direct protein-phosphatase action.

### Wild type and G129E PTEN co-localize and physically interact with AKT

**Figure 8:**
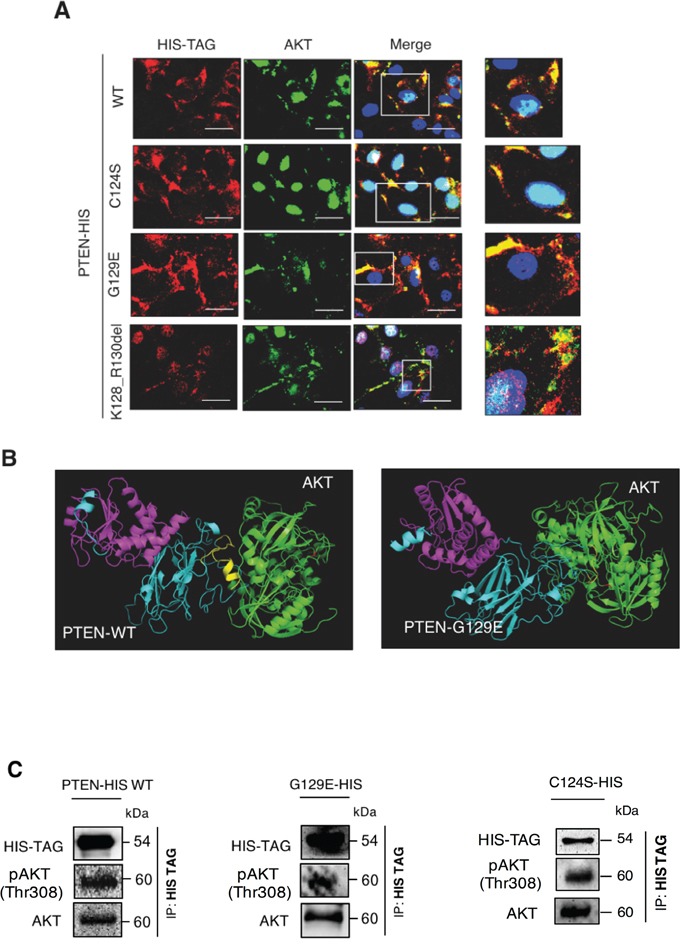
Wild type PTEN and G129E PTEN physically interact with AKT **A.** OVCAR-3 cells adherent onto coverslips were transfected with empty (Sham), WT PTEN, C124S mutant PTEN, G129E mutant PTEN and Deletion 381-393 mutant PTEN HIS-tagged plasmids. The cells were incubated for at least 36 h post-transfection to allow the synthesis of transgenic PTEN, thereafter the cells were fixed in ice-cold methanol and co-stained for HIS-TAG and AKT. DAPI (blue) was used to stain the nuclei. Details of co-localization are shown in the higher magnification images (right side boxes). Scale bar = 20 μM; Magnification = 63X. **B.** Bio-informatic prediction of WT PTEN-AKT and of G129E PTEN-AKT interaction based on ClusPro online software and analyzed with MacPyMOLEdu software. The predicted 3D-structures for PTEN and AKT are colored in light blue and in green, respectively. Relevant domains in PTEN are indicated as follow: phosphatase domain (magentas), C2-terminal domain (blue), the fragment 383-391 (yellow). The phospho-activation (Threonine 308 and Serine 473) of AKT are marked in red. **C.** OVCAR-3 cells were transfected with the HIS-tagged plasmid coding for either PTEN-WT or PTEN G129E. Thirty-six hours post-transfection, the cells were switched to a complete medium or EBSS medium and cultured for 24 h. Finally, the cell lysates were subjected to immunoprcipitation assay with anti-HIS antibody followed by western blotting to check for physical interaction between PTEN and AKT (left panel). The level of expression of the relevant proteins in the cell lysate is shown in the western blotting in the right panel. The experiment was performed three times with similar results. Densitometry analysis in included in the histograms below.

Next, we asked whether PTEN and AKT could interact *in vivo*. First, we performed a fluorescence co-labelling of PTEN and AKT in OVCAR-3 cells transfected with the plasmids coding for the HIS-tagged PTEN isoform. The image data in Figure 8A indicate that AKT co-localizes in the cytoplasm with PTEN, either wt or mutated. Worthy of note, AKT is fully localized in the cytoplasm in the cells expressing the wt or the G129E (protein phosphatase proficient) isoform. Instead, AKT is predominantly found in the nucleus of the cells expressing the C124S PTEN isoform.

We performed a predictive bioinformatic analysis to check if PTEN and AKT have indeed the potential to interact at molecular level. To this end, we used the software ClusPro to check whether the crystallographic structure of wt PTEN (1D5R.1.A) or the predicted structure of G129E PTEN mutant and AKT crystallographic structure (PDB: 3O96) share any interacting domain. As shown in Figure 8B, bioinformatic analysis predicts that wt PTEN and G129E PTEN can interact with AKT, further supporting the co-localization data. To confirm definitively that wt and G129E PTEN are able to interact physically with AKT we performed a co-immunoprecipitation test. To avoid any possible interference with endogenous PTEN, this experiment was performed in the thyroid cancer cell line FTC-133 that is known to be PTEN null and to express high level of phospho-AKT [20]. The cells were transfected with either the wt or the G129E or the C124S PTEN mutant isoforms tagged with HIS. The transgenic PTEN protein was immunoprecipitated with anti-HIS antibody and the immunocomplexes were resolved by SDS-PAGE. Subsequent immunoblotting with specific antibodies revealed the presence of Thr308 phosphoAKT (and of AKT) in the immunoprecipitate of the wt and of both mutant isoforms of PTEN (Figure 8C).

### The wild type and the G129E PTEN, but not the C124S or K128_R130del PTEN mutants, can dephosphorylate AKT

**Figure 9:**
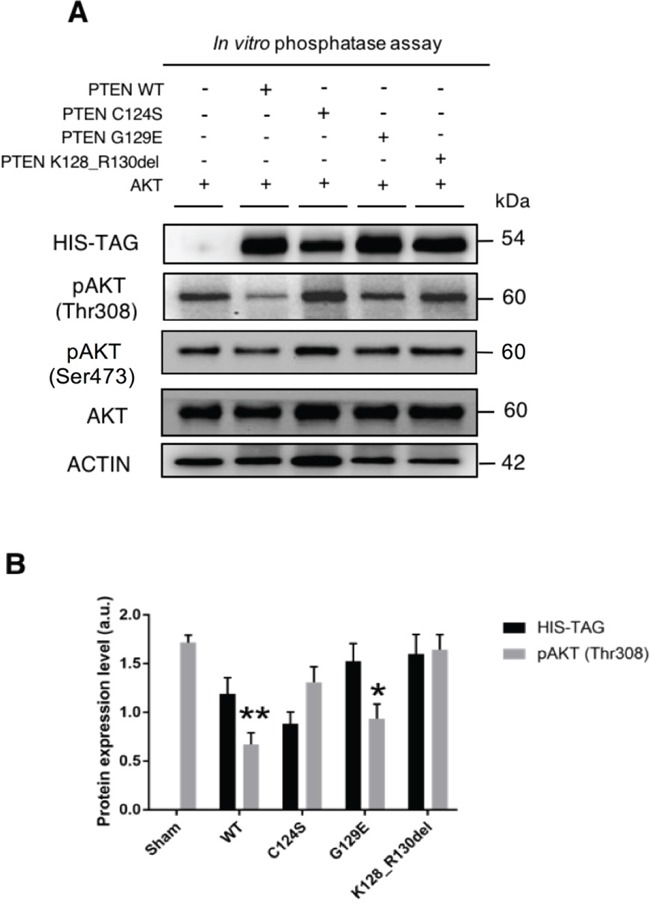
‘*In vitro*’ dephosphorylation of AKT at threonine 308 by PTEN **A.** FTC-133 (PTEN null) cells were transfected with the HIS-tagged plasmid either empty (Sham) or bearing the cDNA for wild type or mutant PTEN. An aliquot of the cell lysate was mixed with the homogenate of OVCAR3 cells expressing phosphorylated AKT. After incubation, the mix was denatured and processed for western blotting. The assay shows that incubation with the cell lysates containing wild-type or G129E mutant PTEN decreases the level of phospho308-AKT in the OVCAR3 sample. The data were reproduced in two separate experiments. **B.** Densitometry quantification of the data in panel A. The level pf pAKT is normalized versus the level of PTEN present in the mix.

Finally, we wanted to prove that PTEN could dephosphorylate AKT. To this end we performed an *ex vivo* assay using the cell homogenates as sources of both the enzyme and the substrate. For the former, we used the PTEN protein eluted from the anti-HIS precipitates obtained from OVCAR-3 cells transfected with either the wt or mutant isoforms of PTEN; and for the latter we used the cell homogenate from FTC-133 cells, which do not express endogenous PTEN and constitutively express phospho-AKT at high level. After incubation *in vitro* of the two components, the mix was resolved by SDS-PAGE and immunoblotted with anti-phospho-AKT antibodies against the Thr308 or the Ser473 sites. The amount of PTEN in the mix was also assessed by immunoblotting with anti-HIS antibody. The results (shown in Figure 9A-9B) demonstrate that both the wt and the G129E PTEN isoforms, but not the C124S and the K128_R130del PTEN mutants, can dephosphorylate AKT at the Thr308 position, while the Ser473 appears slightly dephosphorylated only in the sample incubated with wt PTEN.

### Growth factors limit the protein phosphatase activity of PTEN and its interaction with AKT in cancer cells

**Figure 10:**
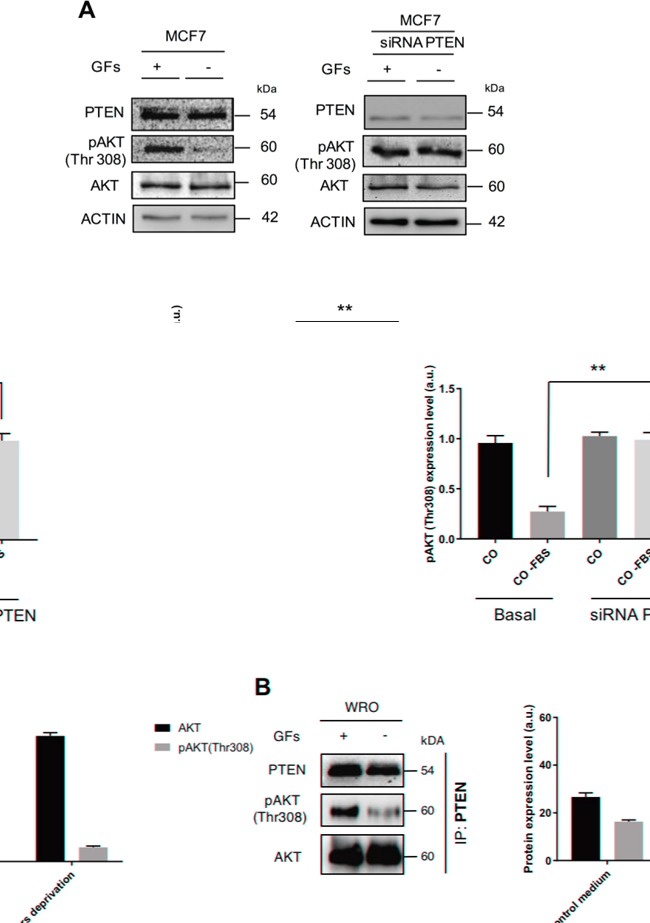
Growth factors affect the interaction of PTEN-AKT and the dephosphorylation of AKT **A.** MCF7 cells were cultured for 24 h in the presence of fetal bovine serum (containing GFs) and thereafter for 4 h in the culture medium devoid of serum. A parallel culture in which PTEN was silenced via siRNA transfection was cultured in the same experimental conditions. Cell homogenates were then assayed by western blotting for the expression of PTEN, AKT and Thr308 phosphoAKT. On removal of GFs AKT was dephosphorylated (left panel), and this effect was not observed in the cells not expressing PTEN (right panel). The experiment was performed in triplicate, and the densitometric values of the relevant bands (average ± S.D.) are shown in the histogram. **B.** WRO cells were cultured for 24 h in the presence of fetal bovine serum (containing GFs) and thereafter for 4 h in the culture medium devoid of serum. At the end, PTEN was immunoprecipitated from the cell homogenates and the presence of AKT and phosphoAKT was assessed in the precipitates by western blotting. The data indicate that a higher amount of AKT and a lower amount of phosphoAKT were bound to PTEN when the cells were cultured in the absence of GFs, compared to the control counterpart. Densitometry of the relevant bands is shown.

Hormones and GFs acting through the PI3KC1 pathway lead to the activation of AKT, which thereafter can elicit the downstream effects as long as it remains phosphorylated. This implies that PTEN should be inactive, at least transitorily. We tested whether the presence of GFs could influence the action of PTEN on AKT. In a first experiment, MCF7 breast cancer cells, which express wt PTEN, were cultured in complete medium (containing GFs) in order to activate the AKT pathway, and thereafter cultured for 4 h in a medium without GFs. In the latter, AKT was found dephosphorylated at position Thr308 (Figure 10A). Analog experiment performed in parallel cultures in which the cells had been transfected with a siRNA to specifically knock-down PTEN indicated that this protein was indeed causing the dephosphorylation of AKT (Figure 10A). Next, we asked whether GFs could interfere with the binding of PTEN to AKT. To this end, we analyzed the immunoprecipitates of PTEN from WRO thyroid cancer cells that had been cultured as above. Strikingly, the precipitate of PTEN from the cells cultured in the presence of GFs contained a lower amount of total AKT and a higher amount of phosphoAKT than those of the respective proteins in the precipitate of PTEN from the cells cultured in the absence of GFs (Figure 10B). Taken together, these data support the view that GFs can limit the PTEN-AKT interaction and the PTEN-mediated dephosphorylation of AKT.

### Bioinformatic analysis of the 3D structure predicts that the K128_R130del PTEN mutant is devoid of both lipid and protein phosphatase activities

**Figure 11:**
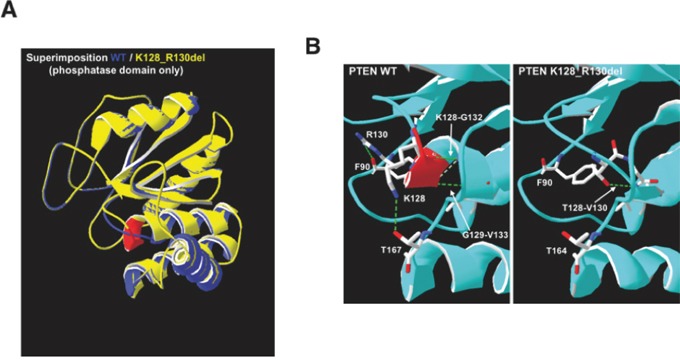
Prediction model of the structural alterations in K128_R130del PTEN mutant **A.** Superimposition of WT (in blue) and K128_R130del (in yellow) PTEN molecules (only the phosphatase domain is shown) performed by Swiss-Model. In red is indicated the tract of alpha helix missing in the PTEN mutant. **B.** Changing in amino acidic bonds between WT and PTEN K128_R130 PTEN in the phosphatase domain. In red is indicated the tract of alpha helix lost in the deleted mutant. The green lines indicate the hydrogen bonds. The analysis was perfomed by Swiss-Model.

The K128_R130del mutation in the exon 5 of PTEN was first described by Saito et al. in A2780 cells. These cells express high level of phospho-AKT, and this effect has been attributed to the loss of the lipid phosphatase activity [35]. Yet, the data here reported are consistent with the hypothesis that the mutation affects also the protein phosphatase function. We used the Swiss-Model to determine the theoretical 3D structure of K128_R130del PTEN in order to check whether the mutation is compatible with the loss of both the lipid and protein phosphatase activities. As shown in Figure 11A, the loss of the three amino acids in the phosphatase domain does not alter the 3D structure of the mutant PTEN. The comparison of the phosphatase domains from wt PTEN and from the mutant PTEN (Figure 11B) reveals a dramatic change in the intra-chain interactions between the amino acids involved in the phosphatase function. In particular, the deletion causes the loss of the following hydrogen bonds: F90-R130, K128-G132, G129-V133, K128-T167; and instead a hydrogen bond between T128 (corresponding to T131 in the wt) and V130 (corresponding to V133 in wt) is formed. Such changes in the coupling of amino acids along with the loss of G129 in the phosphatase domain are likely to affect both the lipid and protein phosphatase function of PTEN.

## DISCUSSION

In order to sustain the anabolic requirements for cell proliferation, cancer cells consume glucose at a rate 30 to 200 times greater than their normal counterparts [2, 37]. The glycolytic rate is even higher in cancer stem cells. A recent study showed that GLUT1-dependent glucose internalization sustains the growth and survival of cancer stem cells, and is essential for the maintenance of the stemness properties [38]. The expression of GLUT1 has been linked functionally to polychemioresistance, cell proliferation and metastasization of tumors [39, 40, 5, 11]. Further, GLUT1 also facilitates the uptake of Vitamin C [41] and of melatonin [42] in cancer cells, and this may have an impact on cancer progression. The above notions underlay the need to improve our knowledge on the pathways that control the expression and translocation of GLUT1 on the plasmamembrane of cancer cells, as this can pave the way for new metabolic therapies [43–45]. The PI3KC1-AKT pathway plays a major role in driving the translocation of GLUT1 from para-golgian vesicles onto the plasmamembrane [20], as also shown in the present study. This pathway is frequently up-regulated in cancer cells because of activating mutations in the PI3KC1 and/or AKT genes. The phosphorylation of AKT relies on the availability of PIP3, which are produced by PI3KC1 and degraded by the lipid phosphatase PTEN. Thus, the lack of functional PTEN also favors the AKT-mediated plasmamembrane expression of GLUT1 and the uptake of glucose [20, 22]. *PTEN* is a tumor suppressor gene very frequently mutated, silenced or deleted in human cancers [46]. This gene codes for a dual lipid and protein phosphatase that influences the behavior and the fate of the cell by regulating the activation of pathways that control the cell metabolism, cell survival and cell death, cell proliferation, cell migration, and genome stability [47, 48, 21]. The most common mutations involving the phosphatase domain (coded by exon 5) of PTEN are C124S [32], G129E [49] and K128_R130del [30], among others. So far, the Y155C PTEN mutant has been described only in a glioblastoma [31]. Here we show that the ovarian cancer cell line OVCAR-3 also expresses this mutant isoform of PTEN.

Besides the intragenic mutations, also epigenetic silencing and post-translational modifications can affect PTEN expression, stability and function [50]. Here we found that PTEN is epigenetically silenced through histone de-acetylation in OAW42 cells. VPA-mediated inhibition of histone de-acetylase, in fact, could rescue PTEN expression, and consequently down-regulate the AKT pathway and glucose uptake in these cells.

The lipid phosphatase activity of PTEN is believed to play the major anti-cancer function, since the inhibition of PIP3-dependent phosphorylation of AKT impacts on a plethora of downstream pathways that control cell proliferation, apoptosis and protein synthesis besides glucose uptake [46]. Besides the lipid-phosphatase activity, PTEN possesses also a tyrosine and serine/threonine phosphatase activity [51]. Yet, the role of the protein-phosphatase activity of PTEN in cancer is largely neglected, also because very few protein substrates involved in the malignant phenotype have been identified so far. PTEN was shown to influence cell migration by dephosphorylating FAK (Focal Adhesion Kinase) [52], chemoresistance by dephosphorylating the non-receptor Tyr kinase SRC [53], and nuclear transcription by dephosphorylating CREB (cAMP responsive-element-binding protein) [54]. More recently, it has been reported that PTEN can dephosphorylate the insulin receptor substrate-1, thus dumping the insulin and Insulin Growth Factor signals that also impinge on glucose metabolism and cell proliferation [55]. Here we show for the first time that PTEN physically interacts with and dephosphorylates AKT. So far, the oncosuppressor function of PTEN has been attributed mainly to its lipid phosphatase activity that antagonizes the activation of the AKT pathway. Our data indicate that PTEN regulates this pathway also through its protein phosphatase activity. In fact, the G129E mutant that lacks the lipid phosphatase activity while retaining the protein phosphatase activity [49] could reduce the level of Trh308-phospho-AKT in the OVCAR-3 cells, which express an active PI3KC1 and an inactive Y155S PTEN mutant, and in the homogenate of FTC-133 cells, which are PTEN null and express constitutively phospho-AKT. The lowest level of phospho-AKT was achieved when the wt PTEN was ectopically expressed in OVCAR-3 cells, consistent with its dual (lipid and protein) phosphatase action in the two steps of the PI3K-AKT pathway, namely at PIP3 level and directly on the Thr308-phospho-AKT. By contrast, the C124S, lacking both the lipid and the protein phosphatase activities [32], and the K128_R130del PTEN mutants were unable to reduce the level of phospho-AKT. The K128_R130del PTEN mutant was first isolated from A2780 cells [30]. This mutation involves the exon 5 in the gene, which codes for the phosphatase domain of the protein. Therefore, not surprisingly, in A2780 cells AKT is highly phosphorylated (Figure 1C) and GLUT1 can be found (at least partly) on the plasmamembrane. In spite of this fact, however, the uptake of glucose in A2780 cells is very limited, probably reflecting the overall low level of expression of GLUT1 protein (Figure 1A). In fact, when this PTEN mutant form is ectopically expressed in OVCAR-3 cells, which express high level of GLUT1 (Figure 1A), the uptake of glucose increases at levels comparable to that in sham transfected cells (Figure 5B). This fact suggests that K128_R130del PTEN is as inactive as the Y155C PTEN endogenously expressed by OVCAR-3 cells. Consistently, in the cells expressing the transgenic K128_R130del PTEN isoform phospho-AKT remains at the same level as in the sham and in the C124S PTEN transfected cells (Figure 6A). Further, the *ex-vivo* phosphatase assay also confirms the inability of K128_R130del PTEN to dephosphorylate Thr308-phospho-AKT, much alike the Y155C PTEN present in the sham-transfected cells and the C124S PTEN (Figure 9A). Taken together, these data lead to the contention that both the Y155C and the K128_R130del PTEN isoforms are devoid of both the lipid and the protein phosphatase activities. The bio-informatics analysis of the phosphatase domain of these mutants is consistent with this conclusion. In fact, in both these mutants a dramatic rearrangement of the interactions among the amino acids of the phosphatase domain without altering the 3D structure of PTEN occurs. This fact indicates that theoretically the PTEN mutants retain the capability to interact with the substrate. Yet, the changes in the amino acids coupling within the phosphatase domain are likely to compromise the lipid and protein phosphatase activity of these mutants, as demonstrated in the present work. What about the normal allele of PTEN in these cells? In A2780 the normal allele is lost [30]. In the case of OVCAR-3 cells, however, the wt exon 5 could be sequenced from the genomic DNA, which indicates that at least a portion of the normal allele is present. We speculate that, however, this allele cannot be fully transcribed. At least two facts argue in favor of this hypothesis: first, OVCAR-3 cells, much alike A2780 cells, constitutively express high level of phospho-AKT even when cultured in the absence of external triggers such as GFs; second, we were not able to isolate the cDNA for the normal allele from OVCAR-3 cells, as well as from A2780 cells. To be noted, our data indicate that GFs limit the protein-phosphatase activity of PTEN and its interaction with AKT. We hypothesize that insulin as well might elicit a transient impairment of PTEN-AKT interaction in order to trigger the downstream signaling.

**Figure 12:**
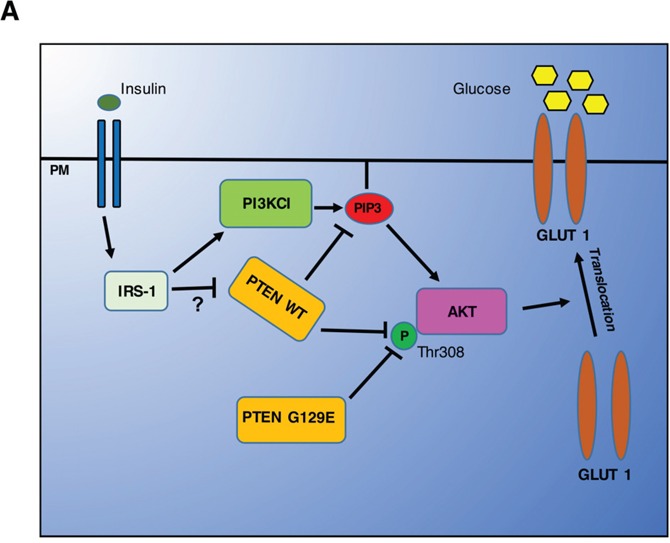
Cartoon illustrating the main findings of the study Insulin and Growth Factors can trigger the phosphorylation of AKT via activation of the PI3KC1 pathway that provides the needed PIP3. Active AKT can then promote the translocation of GLUT1 onto the plasmamembrane to effect the uptake of glucose. PTEN can switch off the AKT pathway by dephosphorylating PIP3, through its lipid-phosphatase activity, and by directly interacting and dephosphorylating AKT at the Thr308 position, through its protein-phosphatase activity. The latter is transiently inhibited in the presence of GFs, and possibly of Insulin as well.

In conclusion, our data emphasize the fact that PTEN acts in two distinct steps of the PI3k/AKT pathway to control the expression of GLUT1 at the plasmamembrane and, further, add AKT to the list of the protein substrates of PTEN (Figure 12). Wild type and mutant isoforms of PTEN were shown equally able to interact with AKT. Interestingly, PTEN dephosphorylated preferentially the Thr308 position, in this resembling the protein phosphatase 2A, whose B55 alpha subunit was shown to selectively target the Thr308 site of AKT [56]. The present study includes the following additional novelties: 1. PTEN is epigenetically silenced in OAW42 cells; 2. OVCAR-3 cells express a Y155C PTEN mutant, and likely present an allelic imbalance with loss of the normal allele; 3. The K128_R130 and the Y155C PTEN mutants, respectively expressed in A2780 and in OVCAR-3 cells, are devoid of both the lipid and protein phosphatase activities.

## MATERIALS AND METHODS

Unless otherwise specified, analytical grade chemicals, cell culture media and supplements were from Sigma-Aldrich.

### Cloning of PTEN cDNA

Total RNA from A2780, SKOV-3, OVCAR-3, and OAW42 cells was purified by TRIzol reagent starting from 1x10^6^ cells following manufacturer's instructions (Thermo Scientific). Total first-strand cDNA was synthesized starting from 1 μg of purified RNA by RT-PCR employing the SuperScript III First-Strand Synthesis System (Thermo Scientific). PTEN cDNA was then cloned by PCR reaction employing the Platinum Pfx DNA polymerase (Thermo Scientific) with the following primers 5’-phosphorylated: *forward* 5’-CATTTCCATCCTGCAGAAGAAG-3’; *reverse* 5’-CCCAATACAGATTCACTTCCTTTAG-3’. PTEN cDNA amplicon (2015 bp length, including the 1295 bp coding sequence and partial 5’ and 3’ UTR) was purified by gel extraction (QIAEXII Gel Extraction Kit, Qiagen) and subject to DNA sequencing by Sanger direct sequencing method employing BigDye Terminator v1.1 Cycle Sequencing kit (Applied Biosystems) following manufacturer's instructions with the following primers: *forward* 271 5’GAAGACCATAACCCACC-3’, *forward* 620 5’-GAACTTGCAATCCTCAGT-3’, *forward* 991 5’-GACAAAGCCAACCGATA-3’; *reverse* 287 5’-GGTGGGTTATGGTCTTC-3’, *reverse* 287 5’-GGTGGGTTATGGTCTTC-3’, *reverse* 633 5’-ACTGAGGATTGCAAGTTC-3’, *reverse* 1007 5’-TATCGGTTGGCTTTGTC-3, *reverse* 1319 5’-CTGGTAATCTGACACAATG’. Cycle sequencing extension products were purified by Ethanol/EDTA/Sodium Acetate precipitation and subjected to automatic sequencing (Applied Biosystems). Primers were form from MWG-BIOTECH AG.

### Cloning of PTEN exon 5

OVCAR-3 total DNA was isolated employing QIAamp DNA Mini Kit (Qiagen) starting from 5x10^6^ cells. PTEN exon 5 was amplified by PCR reaction employing the Platinum Pfx DNA polymerase (Thermo Scientific) with the following primers: *forward* 5’-AAAGCTGGAAAGGGACGAACTGGTG-3’; *reverse* 5’-ACCTGTTTTCCAGGGACTGAGGGTG-3’. Amplicon was purified by gel extraction (QIAEXII Gel Extraction Kit, Qiagen). Purified PTEN exons were then subject to DNA sequencing by Sanger direct sequencing method employing BigDye Terminator v1.1 Cycle Sequencing kit (Applied Biosystems) with the primers employed for the exon 5 cloning. The products of cycle sequencing extension were purified by Ethanol/EDTA/Sodium Acetate precipitation and subjected to automatic sequencing (ABI PRISM 3100, Applied Biosystems). Primers were form from MWG-BIOTECH AG.

### PTEN expression vectors

Wild type PTEN expression vector was constructed by sub-cloning the PTEN cDNA (wild type) from OAW42 cells into the plasmid pcDNA 3.1 Zeo (+) (Thermo Scientific) previously digested with the restriction enzyme EcoRV (New England Biolabs). Wild type Histidine-tagged PTEN [PTEN-(His)_6_] expression vector was generated by 3SM site-directed mutagenesis [57] introducing the sequence codifying for 6 Histidine at the PTEN C-terminus cDNA with the following 5’-phosphorylated primers: *forward* His 5’-**CATCACCATTGA**GGTACCAAGCTTAAGTTTAAACCGC-3’, *reverse* His 5’-**GTGATGGTGACTCGAGCC**GACTTTTGTAATTTGTGTATGCTGATCTTC-3’. The DNA template for this site-directed mutagenesis was the wild type PTEN expression vector.

Mutant PTEN expression vectors codifying for C124S, or G129E, or K128_R130del were generated by 3SM site-directed mutagenesis [56 Follo 2008] with the following 5’-phosphorylated primers: *forward* C124S 5’-**A**GTAAAGCTGGAAAGGGACGAACTGG-3, *reverse* C124S 5’-GTGAATTGCTGCAACAATGATTGTCATC-3’; *forward* G129E 5’-**A**ACGAACTGGTGTAATGATATGTGCATATT-3, *reverse* G129E 5’-CCTTTCCAGCTTTACAGTGAATTGC-3’; *forward* K128_R130del 5’-CTGGTGTAATGATATGT GCATATTTATTA-3’, *reverse* K128_R130del 5’-TTCCA GCTTTACAGTGAATTGCT-3’. The DNA template for this site-directed mutagenesis were the Histidine-tagged PTEN [PTEN-(His)_6_] vector. All of the plasmids were subjected to DNA sequencing to confirm that the appropriate mutation was incorporated and that no additional mutations were present. Primers were form from MWG-BIOTECH AG.

### Cell culture and treatments

All cell lines were maintained under standard culture conditions (37°C, 5% CO_2_). Human ovarian carcinoma cell lines A2780, SKOV-3 and OVCAR-3 were cultured in RPMI 1640 medium supplemented with 10% heat-inactivated fetal bovine serum (FBS) (Euroclone), 1% Glutamine and 1% Penicillin/Streptomycin solution (PES). Human ovarian carcinoma cell line OAW42 was cultured under standard conditions (37°C, 5% CO_2_) in Minimum Essential Medium (MEM) medium supplemented with 10% heat-inactivated FBS, 1% Glutamine, 1% non-essential amino acids and 1% PES. Human follicular thyroid cancer FTC-133 cells were cultured in Dulbecco's Modified Eagle Medium (DMEM)/ nutrient mixture F-12 Ham 1:1 mixture F12, supplemented with FBS (10%), 1% PES and 1% Glutamine. Human follicular thyroid cancer WRO cells were cultured in RPMI 1640 (with L-glutamine) completed by FBS (10%) and PES (1%). Human breast cancer MCF7 cells were cultured in complete DMEM containing 10% FBS and 1% PES.

In serum and amino acids deprivation experiments, the cells were washed thrice with phosphate-buffered saline (PBS) and cultured in Earle's Balanced Salt Solution (EBSS) for the indicated time. Where indicated, the cells were exposed to 5 mM or 10 mM valproic acid sodium salt dissolved in H_2_O.

### Plasmid and siRNA transfections

Histidine-tagged wild type or mutant (C124S, or G129E, or K128_R130del) PTEN cDNAs were transiently expressed by transfection of specific plasmid DNA employing XTREME GENE HD DNA transfection reagent according to the manufacturer's protocol (Roche). As control, transfections with the empty vector (sham) were included. Post-transcriptional gene silencing of PTEN was achieved by Small interference RNA (siRNA). The sequence of PTEN specific siRNA and details on the transfection method have been reported previously [20, 22].

### Multiplex reverse-transcription polymerase chain reaction

A2780, SKOV-3, OVCAR-3, and OAW42 total RNA was purified by TRIzol reagent and total first-strand cDNA was synthesized as described above. Multiplex RT-PCR (34 cycles) was performed according to manufacturer's instructions with Recombinant Taq DNA Polymerase (Thermofisher) starting from 2 μl of cDNA and using a final concentration of 1 μM PTEN primers (*forward* 5’-CATTTCCATCCTGCAGAAGAAG-3’; *reverse* 5’-CCCAATACAGATTCACTTCCTTTAG-3’) and 0,1 μM GAPDH primers (*forward 5’-* TGCACCACCAACTGCTTAGC-3’; *reverse* 5’-GGCA TGGACTGTGGTCATGAG-3’). These conditions were optimized to avoid saturation of the PCR products and were determined in preliminary and separated RT-PCR reactions for each couple of primers. The RT-PCR products were analyzed by agarose gel electrophoresis. DNA ladder was purchased from Fermentas (O’GeneRuler DNA Ladder Mix); primers were from MWG-BIOTECH AG.

### Western blot analysis

Cells were washed twice in cold PBS and harvested with Tris-HCl lysis buffer (Tris-HCl pH 8.5 mM, NP-40 1%, NaCl 150 mM), containing protease and phosphatases inhibitors. Samples were homogenized using an ultrasonic cell distruptor XL (Misonix). Bradford assay was used to measure the protein content. Equal amounts of homogenates (30 μg) were denatured with Leammli sample buffer at 95°C for 5 min, resolved by SDS-PAGE and thereafter blotted onto PVDF membrane (BioRad). The membranes were blocked with 5% non-fat dry milk (Santa Cruz) + 0,1% Tween for 1 h at room temperature. Filters were then incubated with specific primary antibodies overnight at 4°C. This step was followed by incubation with secondary HRP-conjugated antibodies (Goat anti-mouse or Goat anti-rabbit; BioRad) for 1 h at room temperature. The bands were detected using Enhanced Chemiluminescence reagents (ECL; Perkin Elmer) and imaged using the VersaDOC Imaging System (BioRad). For loading control, the filters were stripped and re-probed with β-Tubulin or β-Actin as indicated. Intensity of the bands was estimated by densitometry using ImageJ software (1.48v) from at least three separate experiments.

The following primary antibodies were employed: monoclonal anti-β-Actin (1:2000, A5441, Sigma Aldrich), monoclonal anti-phospho-AKT Thr308 (1:500; #9275, Cell Signaling), monoclonal anti-phospho-AKT Ser473 (1:500; #9271, Cell Signaling), polyclonal anti-AKT (1:500; #4685, Cell Signaling), polyclonal anti-GLUT1 (1:500; #07-1401, Millipore), monoclonal anti-HIS TAG (1:2000, D291-3, MBL), monoclonal anti-PTEN (1:500; #9559, Cell Signaling), polyclonal anti-phospho-P53 Ser15 (1:500; #9284, Cell Signaling), monoclonal anti-P53 (1:200, sc-126, Santa Cruz Biotechnology), monoclonal anti-β-Tubulin (1:2000, T5201, Sigma Aldrich).

### Glucose uptake assay

Cells were seeded on coverslips and treated as indicated. 1 hour before the end of the treatments, 50 μM of fluorescent glucose analogue 2-[N-(7-nitrobenz-2-oxa-1,3-diazol-4-yl) amino] (2-NBDG) was added to each sample and incubated at 37°C. Coverslips were washed three times with PBS, mounted on glass slides and the images acquired immediately with a fluorescence microscope (Leica DMI6000). Fluorescence intensity was kept at minimum to minimize photo-bleaching. Images were taken randomly from at least five different fields.

### Immunocytochemistry

Cells were seeded on coverslips and treated in complete standard medium. Cells were fixed in ice-cold methanol, permeabilized with 0,2% Triton-PBS and then re-fixed with ice-cold methanol. Cells were washed thrice with PBS and incubated overnight at 4°C with indicated primary antibodies dissolved in 0,1% Triton-PBS + 10% FBS. The following primary antibodies were employed: monoclonal anti-AKT (1:100, #4685, Cell Signaling), polyclonal anti-GLUT1 (1:50, 07-1401, Millipore), monoclonal anti-HIS TAG (1:1000, D291-3, MBL), monoclonal anti-PTEN (1:50, sc-7974, Santa Cruz Biotechnology). Cells were washed thrice with PBS and incubated for 1 h at room temperature with indicated secondary antibodies dissolved in 0,1% Triton-PBS + 10% FBS. The following secondary antibodies were employed: IRIS-2 (green fluorescence)- or IRIS-3 (red fluorescence)-conjugated Goat-anti rabbit IgG or Goat-anti mouse IgG (Cyanine Technology). Nuclei were stained with DAPI (4,6-diamidino-2-phenylindole-dihydrochloride). Coverslips were then mounted on glass slides with SlowFade antifade reagent (S36936; Invitrogen) and imaged at 63x magnification with a fluorescence microscope (Leica DMI6000). For immunocytochemistry and cell-live imaging at least five different fields (totally > 50 cells), randomly chosen, were acquired. The acquisition was done with same brightness and contrast setting. The localization of GLUT1 and the internalization of 2-NBDG were determined by phase contrast overlay, cell number was determined by counting nuclei stained with DAPI.

### Co-immunoprecipitation assay

FTC-133 thyroid cancer cells were plated and let adhere 24 h before transfection with the vectors bearing the cDNA for wt, G129E or C124S PTEN. 36 h post-transfection the cells were treated as indicated, washed twice with ice-cold PBS, and harvested with Tris-Hcl buffer (50 mM Tris-HCl pH8), 1% NP-40, 150 mM NaCl) supplemented with phosphatases inhibitors (NaV, NaF) and proteases inhibitor cocktail (1μg/μl, Sigma). In each plate, 15 min before the ending of the treatment, 0.5 M of the chemical cross-linker 3-3’-dithiodipropionic acid di-(N-hydroxysuccinimide ester) (Sigma Aldrich) dissolved in DMSO was added. The same amount of protein (400-500 μg) was incubated with anti-HIS TAG antibody (2 μg) for at least 1 h at 4°C under rotation. In the case of the WRO thyroid cancer cells, which express endogenous wt PTEN, the cells were incubated with the cross-linker as above and the cell homogenate precipitated with anti-PTEN. To capture the immunocomplex, 50 μl of sepharose G beads (P3296, Sigma) were added to each sample and left under rotation overnight at 4°C. Immunocomplexes were then precipitated by centrifugation (1000 g) and eluted with 80 μl of Leammli buffer 1x at 95°C for 10 min. Equal volume of eluate was loaded on a SDS-containing polyacrylamide gels and immunoblotted with specific antibodies to reveal the presence of AKT and phosphoAKT in the immunoprecipitates.

### Phosphatase assay

FTC-133 cell homogenate was employed as substrate homogenate because of the high levels of phosphorylated AKT (Thr308) and lack of PTEN. Cells were harvested with Tris-HCl lysis buffer supplemented with proteases and phosphatases inhibitors. OVCAR-3 cells were transfected with histidine-tagged expression vectors bearing the cDNA for wild type or mutants PTEN. PTEN was immunoprecipitated from individual OVCAR-3 cell homogenate employing an anti-HIS-TAG antibody. Immunocomplexes were eluted with Leammli buffer 1X in a final volume of 30-50 μl.

30 μg of FTC-133 cell homogenates were incubated with equal amount of immunoprecipitated PTEN. Phosphatase reactions were carried out for 15 min at 37° C. As control, Leammli buffer 1X was added in one tube containing 30 μg of FTC-133 total lysate and kept at 37°C for 15 min. At the end of the reaction, samples were boiled at 95°C in Leammli buffer 5X and then separated by SDS-PAGE. Immunoblottings were developed using antibodies against HIS-Tag, pAKT (Thr308) and total AKT.

### Bioinformatic analysis

The theoretical 3D structure of PTEN WT and mutants Y155C and K128_R130del was determined with Swiss-Model (www.expasy.ch) using the wild type PTEN crystallographic structure 1D5R.1.A [58] as template. The superimposition of the predicted 3D structure of Y155C mutant PTEN with wild type PTEN structure 1D5R.1.A and of phosphatase domains of WT and K128_R130del were made with Swiss-Pdb viewer (Glaxo Smith Kline). The predicted interaction between wild type PTEN structure 1D5R.1.A or its mutants predicted 3D structures with AKT crystallographic structure (Pdb code:3O96) was performed with ClusPro (www.cluspro.bu.edu). Predicted interaction data were imaged and analyzed with MacPyMOLEdu software (v1.3).

### Statistical analysis

All data refer to at least three separate experiments performed by different operators. Histograms are shown as mean + S.E.M. and were considered statistically significant with p value < 0,05 (Unpaired *t* test, two-tail). * = p < 0,05; ** = p < 0,01; NS = no statistically significant.
